# Biochemical and Ultrastructural Changes in *Sida cordifolia* L. and *Catharanthus roseus* L. to Auto Pollution

**DOI:** 10.1155/2014/263092

**Published:** 2014-10-30

**Authors:** Vijeta Verma, Neelam Chandra

**Affiliations:** Department of Botany, University of Lucknow, Lucknow 226007, India

## Abstract

Auto pollution is the by-product of our mechanized mobility, which adversely affects both plant and human life. However, plants growing in the urban locations provide a great respite to us from the brunt of auto pollution by absorbing the pollutants at their foliar surface. Foliar surface configuration and biochemical changes in plant species, namely, *Sida cordifolia* L. and *Catharanthus roseus* L. grown at roadside (polluted site 1, Talkatora; polluted site 2, Charbagh) in Lucknow city and in the garden of the university campus, which has been taken as reference site, were investigated. It was observed that air pollution caused by auto exhaust showed marked alterations in photosynthetic pigments (chlorophyll, carotenoid, and phaeophytin), and relative water content was reduced while antioxidative enzymes like catalase and peroxidase were found to be enhanced. The changes in the foliar configuration reveal marked alteration in epidermal traits, with decreased number of stomata, stomatal indices, and epidermal cells per unit area, while length and breadth of stomata and epidermal cells were found to be increased in leaves samples wich can be used as biomarkers of auto pollution.

## 1. Introduction

Air pollution, a global problem being faced by both the developed nations and the developing ones, has been aggravated by developments that typically occur as countries become industrialized: growing cities, increased traffic, rapid economic development and industrialization, and high levels of energy consumption. Air pollution is a major problem arising mainly from industrialization [1]. Motor vehicles account for 60–70% of the pollution found in an urban environment [2–4]. It is estimated that vehicles account for 70% of CO, 50% of HC, 30–40% of NOx, 30% of SPM, and 10% of SO_2_ of the total pollution load in the major metros of India, of which two-thirds are contributed by two wheelers alone [5, 6].

Besides the human and animal populations, this problem has drastic impacts on the local environment and causes extensive damage to vegetation including crops, fruit trees, medicinal plants, and ornamentals. Plants being directly and constantly exposed to the pollutants (both gaseous and particulates) play a significant role as indicators and in mitigating the problem. They absorb, accumulate, and integrate the pollutants impinging on their foliar surface, acting as the sinks for various pollutants and thus mitigating the problem. The plants do not render this service to mankind without any serious implications; in turn, they suffer from various deformities caused by the integrating pollutants and show diverse morphological, biochemical, anatomical, and physiological responses. Thus, the plants can be used as both passive biomonitors and biomitigators in the urban environment to indicate the environmental quality and to attenuate the pollution level in a locality [7, 8]. Pollutants like SO_2_, O_3_, and CO_2_ have many harmful effects as they affect the physiological activities of the plants. Sulfur dioxide is responsible for the increase in oxidative stress. They enhanced the production of reactive oxygen species like H_2_O_2_ which is very harmful to the metabolic activities of the plants [9]. Sulfur dioxide polluted regions showed the higher activities of peroxidase [10, 11]. Sulfur dioxide also affects the process of photosynthesis [12]. Vehicular traffic is the main source of particulate air pollution in Lucknow and these are the prime source of air pollution in urban areas [13]. The number of different categories of vehicles registered with RTO (Regional Transport Office), Lucknow, is 12,09,745 on March 31, 2011, which is 9.23% higher over the last year. Charbagh is the most polluted area in Lucknow, says a report published by Indian Institute of Toxicology Research. The study carried out in three locations of the state capital showed that Charbagh has the highest average level of respirable particulate matter (RSPM), sulfur dioxide, and oxides of nitrogen among all the locations. As per the national ambient air quality standards (NAAQS), Charbagh had RSPM of 253.66 with 23.62 and 58.42 micrograms per cubic meter of sulfur dioxide and oxides of nitrogen, respectively. The report cited the addition of 109,773 vehicles on the roads of Lucknow as the chief reason for pollution and called for emergency attention of policy markers, researchers, and regulatory agencies.

*Sida cordifolia* L. is an erect perennial commonly known as flannel weed. Flannel weed is used in Ayurvedic medicine. It is used in neurological ailments, especially in stroke rehabilitation. It has been investigated as an anti-inflammatory for preventing cell proliferation and for encouraging liver regrowth. Due to its ephedrine content, it possesses psychostimulant properties, affecting the central nervous system and also the heart. The roots and stems contain the alkaloids ephedrine.* Catharanthus roseus* L., also known as periwinkle, is an important medicinal plant which belongs to family Apocynaceae and is an important source of indole alkaloids which are present in all plant parts. Periwinkle is used for the treatment of diabetes, fever, malaria, throat infections, and chest complaints. It is also used for the regulation of menstrual cycles and as a euphoriant. The physiologically important and antineoplastic alkaloids, namely, Vincristine and Vinblastine, are mainly present in the leaves, whereas antihypertensive alkaloids, such as ajmalicine, serpentine, and reserpine, are reported to be present in the roots. Vincristine and Vinblastine alkaloids are used in the treatment of various types of lymphoma and leukemia. These* Catharanthus* alkaloids are also used for the treatment of both malignant and nonmalignant diseases and in platelet and platelet associated disorders. Both plants are used as roadside plants as a green belt in cities. In view of plants' role in the indication and abatement of air pollution, this study was carried out to assess the impact of automobile pollution on the plant metabolism and foliar surface configuration of plants growing along the roadsides in the urban locality in order to identify their role as the biomarkers of air pollution.

## 2. Materials and Methods

For micromorphological and physiological studies, leaves of* Sida cordifolia* L. (flannel weed) and* Catharanthus roseus* L. (periwinkle) were collected from the Botany Department, Lucknow University, which was found to be healthy and treated as control (C) and also the plants growing inside the Talkatora (Industrial area P1) which has been taken as polluted site (industrial area, P1), Charbagh, loco workshop, P2, which has been taken as polluted site. For micromorphological evaluation, whole studies were carried out by comparing only one polluted site (P2) with healthy site in the summer. The pieces of leaves were cut (1 × 1 cm^2^) and placed in maceration mixture (conc. HNO_3_ + H_2_CrO_3_ in a ratio of 9 : 1). Cuticles were stained in sudan 1 V and mounted in glycerin jelly. Epidermal structures were examined under low and high power of the microscope. Terminology used was the same as suggested by Pant [14], Stace [15], and Dilcher [16]. Scanning electron microscopy (SEM) examination of values of both genera was carried out at the Birbal Sahni Institute of Paleobotany (BSIP), Lucknow, India.

For biochemical and physiological studies, both polluted sites (P1 and P2) were taken into consideration in comparison to healthy site in both the summer and the winter. Photosynthetic pigments in leaves were extracted in 80% acetone by the method of Lichtenthaler et al. [17]. The extract was centrifuged and read out at 480 and 510 nm for carotenoid, 645 and 663 nm for chlorophyll, and 655 and 666 for phaeophytin. Catalase was assayed by the method of Euler and Josephson [18]. The reaction mixture for enzyme assay contained 0.005 M hydrogen peroxide in 0.025 potassium phosphate buffer pH 7.0 and this was standardized against 0.1 N KMnO_4_. Suitable amount of aliquot was added. After 5 minutes, the reaction was stopped by adding 2 N H_2_SO_4_. Peroxidase was estimated by the modified method of Luck et al. [19]. To 2 mL of 0.1 M phosphate buffer pH 6.0, 0.01% H_2_O_2_ and 0.5% (w/v) p-phenylenediamine were added. Reaction was stopped by adding 4 N H_2_SO_4_. The whole reaction was carried out at 25°C and color intensity was read at 485 nm.

### 2.1. Statistical Analysis

The data has been presented in tables and figures and is the mean of the observations made in triplicate (*n* = 3) along with the standard error and the least significant differences between mean values (*P* ≤ 0.05). The data has been put to ANOVA.

## 3. Result and Discussion

### 3.1. Micromorphological Structures

**Figure 1:**
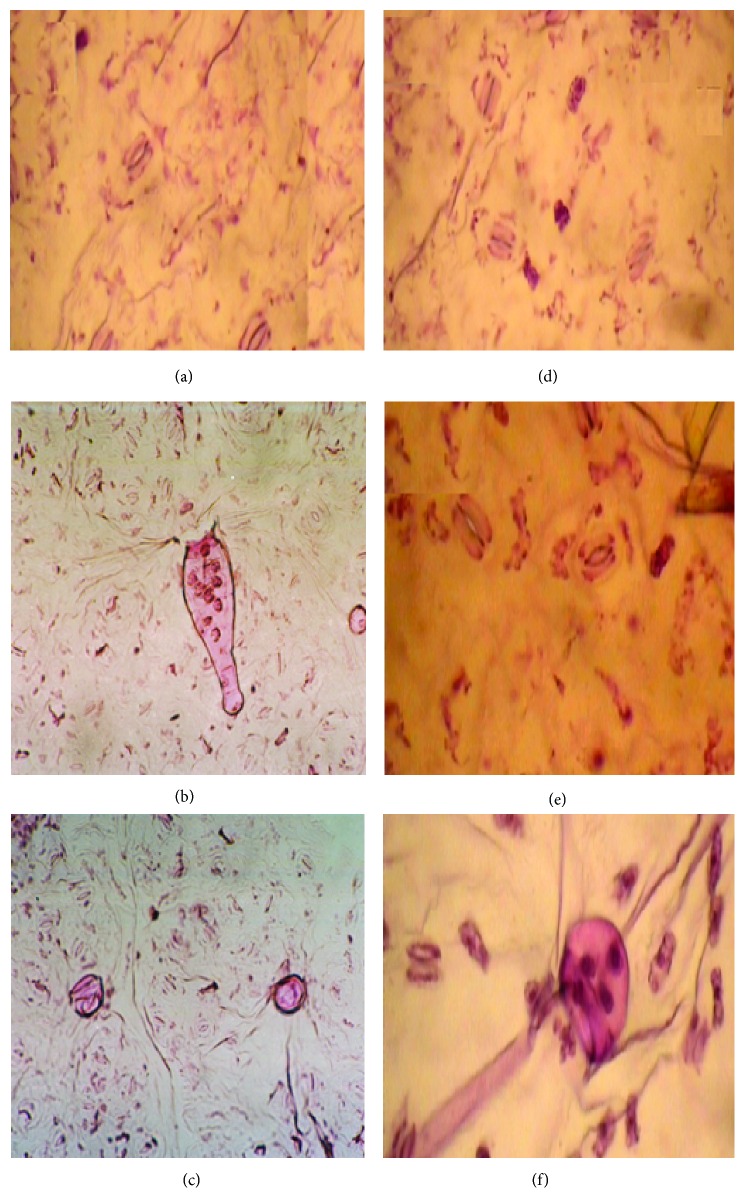
Effect of air pollution on micromorphological structures on leaves of* Sida cordifolia* L. in the summer (left side: healthy site; right side: polluted site 2) ((a) upper foliar surface showing stomata; (b) lower foliar surface showing stomata with striations at the trichome base; (c) lower foliar surface showing distribution of stomata with developing trichome base; (d) upper foliar surface showing slit-like aperture of stomata; (e) lower foliar surface showing distribution of stomata; (f) lower foliar surface with degenerating trichome).

**Figure 2:**
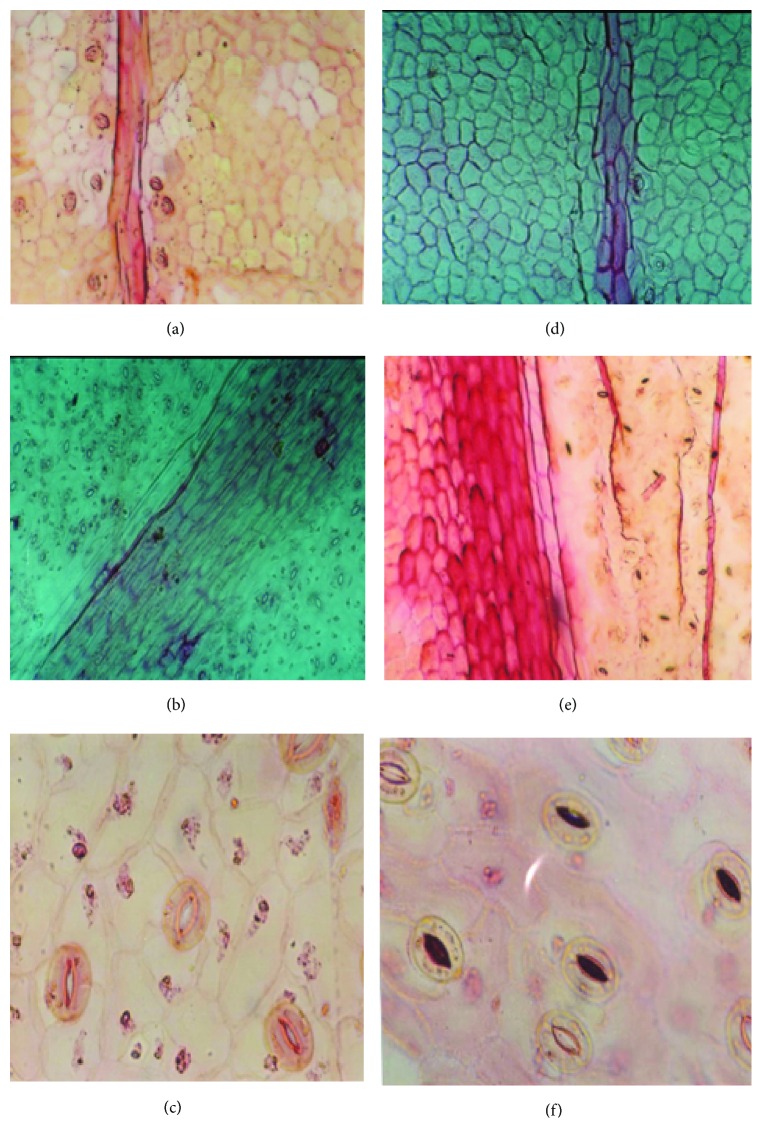
Effect of air pollution on micromorphological structures on leaves of* Catharanthus roseus* L. in the summer (left side: control; right side: polluted site 2). ((a) upper foliar surface showing stomata on either side of midvein; (b) lower foliar surface showing stomata with thin walled marginal cells; (c) lower foliar surface showing epidermal cells with stomata; (d) upper foliar surface showing stomata on one side of midvein; (e) lower foliar surface showing occluded stomata with thick walled marginal cells; (f) lower foliar surface showing occluded stomata).

**Figure 3:**
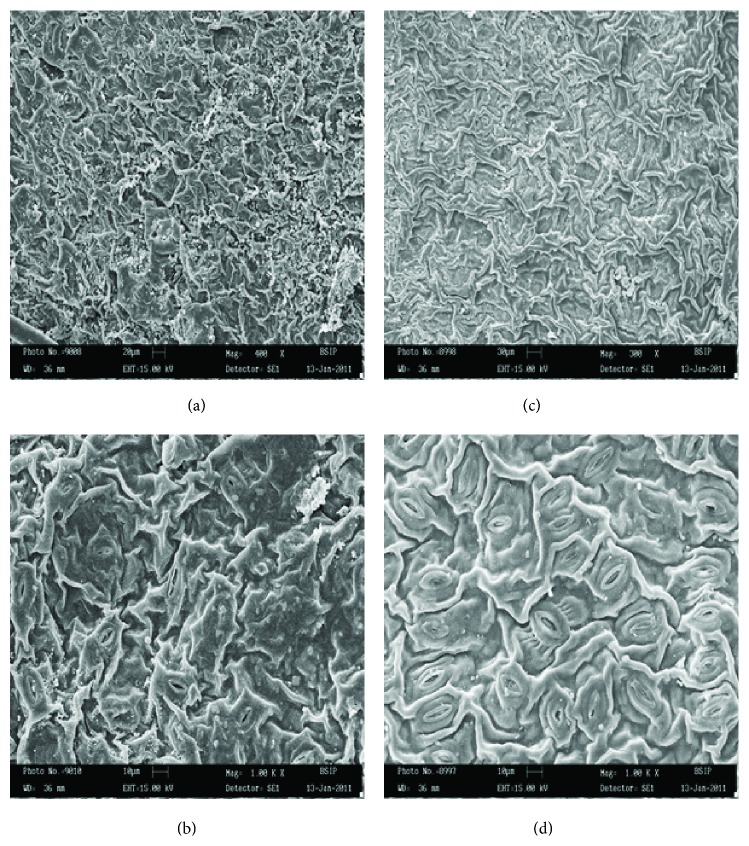
SEM photographs showing the effect of air pollution on leaf surface of* Sida cordifolia* L. (left: (a) = lower surface showing sunken stomata; (b) = lower foliar surface showing distribution of stomata with occluded stomatal pore) and* Catharanthus roseus* L. (right: (c) = upper foliar surface showing encrypted and sunken stomata; (d) = lower foliar surface showing stomata with striations arising from subsidiary cells) collected from Charbagh in the summer.

The use of plants as monitors of air pollution has long been accepted since plants often are the initial indicators of air pollution. Individual plant responds differently to different air pollutants. Plants improve the quality of urban life due to their large leaf areas, relative to the ground on which they stand. Depending on structural properties of their surface, they can act as biological absorbers or filters of pollutants [7]. In this way, they remove huge quantities of gaseous pollutants and airborne particles, thus improving the quality of the environment. Some plant species have been identified to be able to absorb, detoxify, and tolerate high levels of pollution [20]. The foliar surface is the most important receptor of atmospheric pollutants where they cause several structural and biochemical changes. The present studies on the plants growing in loco area indicate that auto exhaust pollution brought appreciable changes in the number of epidermal cells and stomata per unit area. Plants of both genera growing very close to the loco area showed an adverse effect on this number. In the present investigation, it has been observed that the stomatal and epidermal frequencies decline, resulting in the significant fall in stomatal index (Table 1). Size of epidermal cells and stomata was increased in polluted population. Since differentiation of stomata mother cells requires division of epidermal cells, therefore, a decrease in the frequency of stomata should be normally accompanied by an increase in the size of epidermal cells. Our results were in conformity with the findings of [21] which studied the effect of environmental pollution on morphology and leaf epidermis of* Commelina bengalensis*. Results were the same; stem length, leaf area, flower size, and fruit size showed a marked reduction in growth in the plants from the polluted area. Length and density of trichome increased, while stomatal frequency values and the epidermal frequency decreased, in response to environmental pollution (Table 1). Plants of flannel weed showed changes in their leaf morphology due to environmental stresses. Lower foliar surface showed stomata with board aperture and large size trichome, while slit-like aperture of stomata was observed in polluted site plant in comparison to control (Figure 1). It has been reported that air pollutants increase cell permeability by damaging the membrane integrity [22]. Pollutant induced increased cell permeability may cause a loss of water from the guard cells to make them flaccid, which result in stomatal closure. In* Catharanthus roseus* L. stomata was observed on either side of mid vein on the upper surface of healthy population [Figure 2(a)], but stomata on one side of mid vein [Figure 2(d)] was observed on the plants collected from highly polluted site. Lower foliar surface showed stomata with thin walled marginal cells (Figure 2(b)) in healthy plants, while, in polluted population, there were thick walled marginal cells with occluded stomata (Figure 1(e)) observed. Morphological characters of plants are very important in determining plant resistance to air pollution. Pal et al. [23] studied sunken stomata and thick cuticles; small and dense cells and suberised cell walls are in favor of reducing pollutant entry into leaves and cells. When the leaf samples of these plants were examined under SEM, the following observations were recorded. SEM micrographs of lower foliar surface of flannel weed showed sunken stomata with occluded stomatal pores. It was also observed that cuticle was ruptured at the edge of stomata to form a crypt-like structure (Figure 3). In SEM micrographs of periwinkle, upper foliar surface showed encrypted and sunken stomata, while lower foliar surface showed stomata with striations arising from subsidiary cells (Figure 3).

### 3.2. Chlorophylls

**Figure 4:**
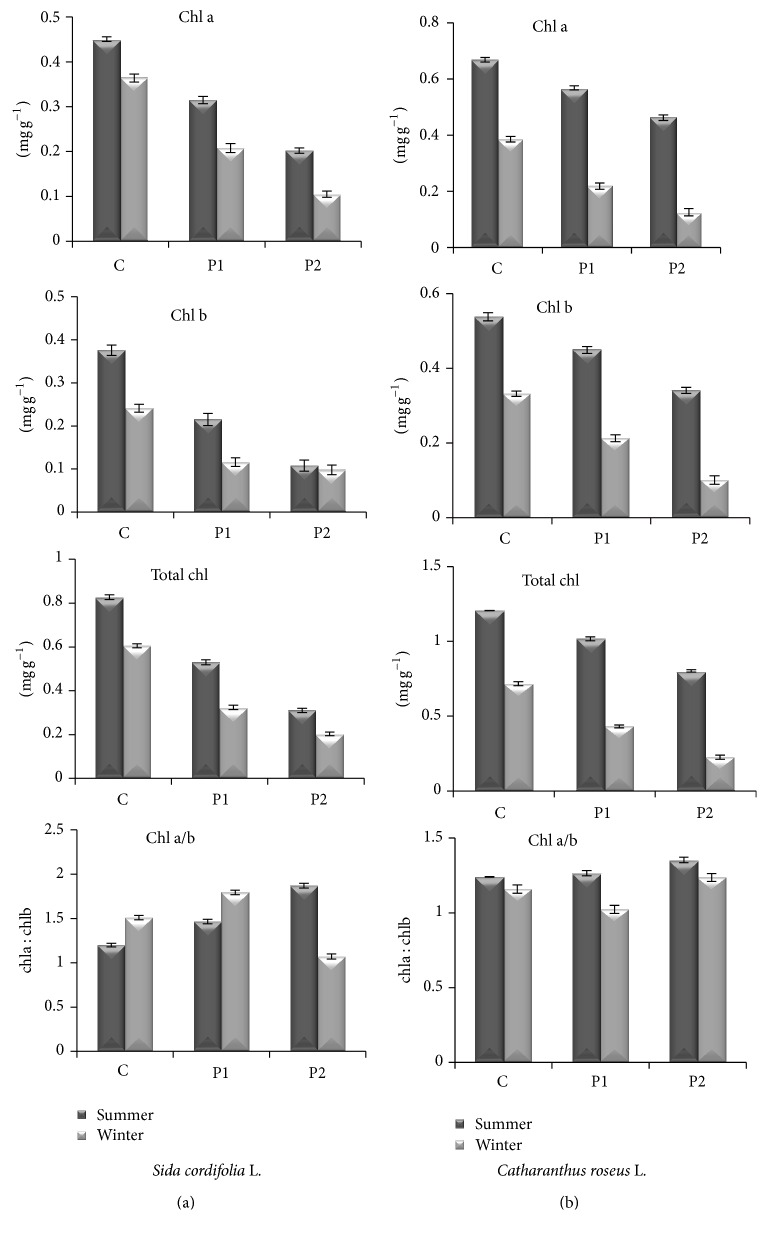
Effect of auto pollution on chlorophylls (chl a, chl b, total chl, and ratio of chl a and chl b) in leaves of* Sida cordifolia* L. (a) and* Catharanthus roseus* L. (b) (C = healthy site; P1 = polluted site 1; P2 = polluted site 2). Data represent mean ± S.E. (*P* ≤ 0.05).

Chlorophylls a and b both were found to be decreased in leaves of both plants collected from polluted site in comparison to plants collected from healthy site (HS) healthy site (Figure 4). The concentration of chl a and chl b content in flannel weed in the summer at polluted site (P1) was decreased up to 30% and 43%, while at polluted site 2 (P2) it was found up to 55% and 71% in comparison to healthy site. In the winter, chlorophyll a was decreased to 43% and 71% at P1 and P2, respectively. In the winter, chlorophyll b also showed a reduction of 52% and 59% at P1 and P2, respectively, in comparison to healthy site. Total chlorophyll was highly reduced at P2 in comparison to P1 in both seasons (Figure 4). Ratio of chl a and chl b was found to be increased in the summer at both sites. But in the winter, ratio of chl a and b was found to be increased (19%) at P1 and decreased (29%) at P2 in comparison to healthy site. In periwinkle, chl a was highly reduced in the winter at both P1 (43%) and P2 (67%) in comparison to healthy site, while in the summer it was decreased up to 15% and 31% at P1 and P2 sites in comparison to healthy site. In periwinkle, total chlorophyll was also reduced in plants collected from P1 and P2 in comparison to healthy site, and reduction was more pronounced in the winter. Ratio of chl a and chl b was increased in both seasons at both sites except in the winter at P1 site where it was found to be decreased (Figure 4). Chlorophyll measurement is an important tool to evaluate the effect of air pollutants on plants as it plays an important role in plant metabolism, and any reduction in chlorophyll content corresponds directly to plant growth [24]. Leaf chlorophyll content, thus, can provide valuable information about physiological status of plants. Pollutants like SO_2_ entering the leaf are hydrated to form HSO_3_, SO_3_, and H+ ions. Sulfite affects the carbon fixation, ribulose biphosphate carboxylase, glycolate oxidase activity, and photophosphorylation [25]. Increasing level of air pollution in Lucknow is a major contributor to the formation of dense fog and low day temperature in winters. Suspended particle matter (SPM) emitted by vehicles and in the air combines with the moisture content in the atmosphere to increase the density of the fog, which lasts for a longer period. The SPM are present in the atmosphere in summers as well but do not pose much problem because moisture content in the atmosphere is very low. In monsoon season, the pollutants are cleared by rainfall. However, in winters, as temperatures come down due to icy winds coming to plains from the snow clad hills, the moisture content is condensed into fog. The presence of SPM expedites the process of fog formation, leading to a drop in day temperatures and chilly weather. The fog thus formed bars penetration of sun rays leading to a fall in temperature during the daytime. A Higher chlorophyll content was found in the monsoon season than in the summer and is the least in the winter; it might be due to the high NO_2_ concentration which increases the sensitivity of plant to winter stress as observed by Swarnlata et al. [26]. Low-chlorophyll-content winter is due to the high pollution level, temperature stress, low sunlight intensity, and short photoperiod.

Present study revealed that chlorophyll content in both plants varies with the pollution status of the area; that is, the higher the pollution level in the form of automobile exhaust the lower the chlorophyll content. It also varies with the tolerance as well as sensitivity of the plant species. Studies [3, 27] also suggest that high levels of automobile pollution decrease chlorophyll content in plants near roadsides. The reason for degradation of chlorophyll pigments can also be attributed to the action of SO_2_ and NO_2_ on the metabolism of chlorophyll [28]; both of these gases are the constituents of vehicular emissions. The reduction in the concentration of chlorophyll might have also been caused by the increase in chlorophyllase enzyme activities, which, in turn, affects the chlorophyll concentration in plants [29]. Our results are also in agreement with the work of [30].

### 3.3. Carotenoids and Phaeophytin

**Figure 5:**
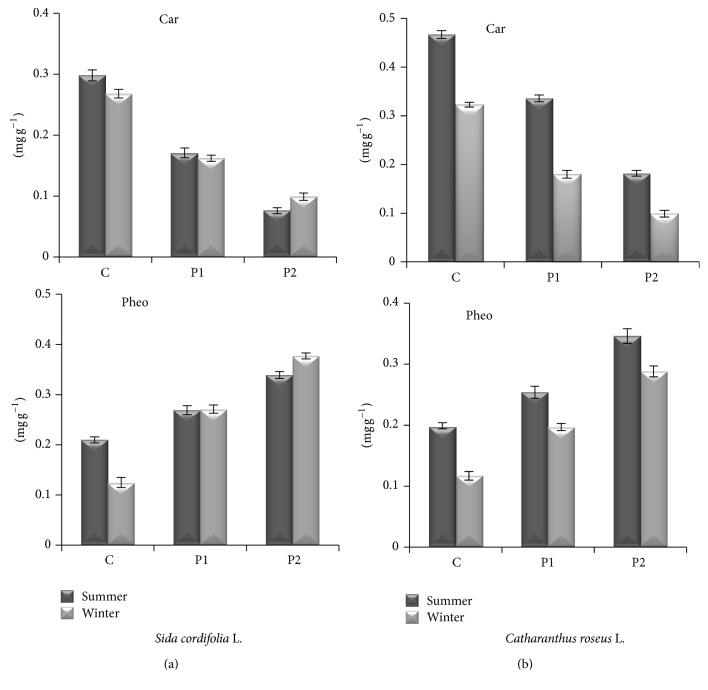
Effect of auto pollution on carotenoids (car) and phaeophytin (Pheo) in leaves of* Sida cordifolia* L. (a) and* Catharanthus roseus* L. (b) (C = healthy site; P1 = polluted site 1; P2 = polluted site 2). Data represent mean ± S.E. (*P* ≤ 0.05).

In both genera, carotenoids were found to be reduced in both seasons at both sites in comparison to healthy site but phaeophytin was found to be increased (Figure 5). In flannel weed, Decrease in carotenoids concentration was more in the winter at healthy site. Percentage decrease at P1 was marginally increased in the summer in comparison to the winter. At P2 carotenoids concentration was highly reduced (up to 74%) and the reduction was more pronounced in the summer. In periwinkle, carotenoids concentration was also reduced in both seasons and both sites. Reduction was significantly high in the winter at P2 in comparison to healthy site (Figure 5). Agarwal and Sharma [31] have reported significant reduction in carotenoids in leaf samples collected from polluted environment. Aquil et al. [32] have also reported a reduction in carotenoids content in the plants of* Albizzia lebbek* Benth due to being exposed to coal smoke. Carotenoids protect photosynthetic organisms from potentially harmful photooxidative processes and are essential structural components of the photosynthetic antenna and reaction center [33]. The pheophytin concentration of plant leaves of both genera was found to be opposite that of chlorophyll concentration. Higher level of pheophytin was found in the winter in both the plant species when the pollution level was high. In flannel weed and periwinkle, the highest concentration of phaeophytin was observed in the winter at P2 but the increase was more (202%) pronounced in flannel weed (Figure 5). It might be due to the breakdown of chlorophyll to pheophytin as suggested by Rao and Le Blanc [34]. It indicates the sensitivity of plants to the pollutants; the higher the phaeophytin level in the plant is, the greater the sensitivity to air pollution is.

### 3.4. CAT, POD, and RWC

**Figure 6:**
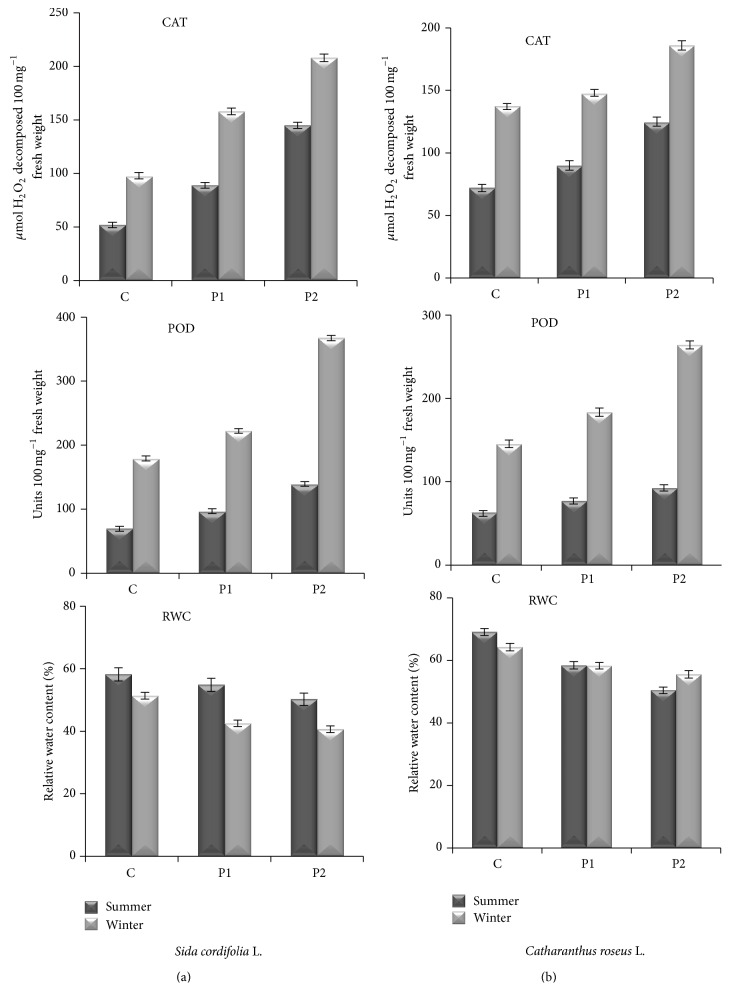
Effect of auto pollution on catalase (CAT), peroxidase (POD), and relative water content (RWC) in leaves of* Sida cordifolia* L. (a) and* Catharanthus roseus* L. (b) (C = healthy site; P1 = polluted site 1; P2 = polluted site 2). Data represent mean ± S.E. (*P* ≤ 0.05).

The activity of CAT at both sites in both seasons in flannel weed and periwinkle was found to be increased (Figure 6). Maximum increment in CAT activity in flannel weed and periwinkle was 112% and 36%, which was observed at P2 site in the winter. In the summer, maximum (179%) increment in CAT activity was observed at P2 in flannel weed. Peroxidase activity was more in the winter than in the summer at healthy site (Figure 6). In the winter, the percentage increase in POD activity in flannel weed. In periwinkle POD activity was found to be 24% and 26% at P1 and 105% and 81% at P2, respectively. In the summer, the percentage increase in POD activity in flannel weed and periwinkle* was* found to be 40% and 24% at P1 and 101% and 49% at P2, respectively. Antioxidative enzymes CAT and POD are effective quenchers of reactive oxygen species (ROS) and play an important role in the adaptation and ultimate survival of plants during periods of stress. As plants produce significant amount of antioxidants to prevent oxidative stress caused by photons and oxygen, they represent a potential source of new compounds with antioxidant activity. The activity of CAT and POD increases under the air pollution. Studies of Karjalainen et al. [35] suggested that POD is a specific indication of SO_2_ and NO_2_ pollution. In combination, SO_2_ and NO_2_ induced strong POD activity in plants. Basically, gaseous pollutants like SO_2_ are taken up by stomata and solubilized in aqueous phase of the cell wall and can diffuse across the plasma membrane. Sulfur dioxide is oxidized in aqueous phase by producing HSO_3_^−^ and H^+^ ions. Peroxidases have a very important role as they may act as sulfite oxidases in the presence of monophenolics [11].

Relative water content (RWC) represents a useful indicator of the status of water balance of a plant, essentially because it expresses the absolute amount of water, that the plant requires to reach artificial full saturation (Gonzalez-Vilar, 2001) [36]. Relative water content was found to be low in polluted plants in comparison to healthy plants (Figure 6). Air pollution affects the RWC of leaves very effectively. More RWC of a leaf helps to maintain its physiological balance under stress condition of dust pollutants. Higher RWC also favors drought resistance. The relative water content in the leaves of flannel weed at P1 showed reduction of 6% and 14%, while in periwinkle, the reduction was 15% and 27% at P2 in the summer in comparison to healthy site. Significant reduction was observed in flannel weed (21%) and in periwinkle (14%) in the winter at P2 in comparison to healthy site (Figure 6). It was observed that RWC declines more in sensitive species than that in tolerant ones, as reported by Singh [37]. It is evident from the above discussion that the pollutants such as SPM, SO_2_, and NO_2_ from automobile exhaust not only cause bad air quality conditions around nearby areas but also cause significant reduction in morphological and physiological parameters.

Present studies clearly indicate that the vehicular induced air pollution reduces the concentration of photosynthetic pigments (chl a, chl b, carotenoid, and phaeophytin) in both the species exposed to roadside pollution. The antioxidative enzymes (catalase and peroxidase) were found to be enhanced while relative water content was reduced. In micromorphological studies, there was a reduction in stomatal frequency, epidermal cell frequency, and stomatal index, while length and breadth of stomata and epidermal cells were found to be increased in both the species. Marked alterations were observed both in the physiological status and in the foliar surface ultrastructural configuration of both periwinkle and flannel weed plants growing at highly polluted site in comparison to healthy site. Therefore, these plant species may be used as biomarkers and mitigators of pollutants coming out of the automobile exhaust. Significant changes were recorded in flannel weed in comparison to periwinkle in the studied parameters. It was noticed from the above result that periwinkle was found to be more tolerant compared to the flannel weed.

## Figures and Tables

**Table 1:** Effect of air pollution on the micromorphological structures present on the leaves of *Sida cordifolia* L. and *Catharanthus roseus* L. collected in the summer.

Plant name	Micromorphological structures	Parameters	Lucknow University (nonpolluted area, HS)		Charbagh (polluted site, P2)	
			Abaxial surface	Adaxial surface	Abaxial surface	Adaxial surface
*Sida cordifolia* L.	Epidermal cells	Frequency	175 ± 1.675	140 ± 1.989	^*^152 ± 1.543	^*^118 ± 1.833
		Size in *µ*m	*L*—12 ± 0.489	*L*—11 ± 0.324	^*^ *L*—20 ± 0.571	^*^ *L*—18 ± 0.110
			*B*—07 ± 0.421	*B*—06 ± 0.211	^*^ *B*—13 ± 0.114	^*^ *B*—13 ± 0.098
	Stomata	Frequency	20 ± 0.523	15 ± 0.760	^*^11 ± 0.212	^*^07 ± 0.311
		Size in *µ*m	*L*—06 ± 0.214	*L*—04 ± 0.214	*L*—08 ± 0.214	*L*—06 ± 0.189
			*B*—02 ± 0.115	*B*—02 ± 0.115	^*^ *B*—05 ± 0.115	*B*—04 ± 0.097
		Stomatal index	14 ± 0.117	10 ± 0.387	^*^06 ± 0.241	^*^04 ± 0.265
	Trichomes	Frequency	05 ± 0.126	03 ± 0.056	^*^09 ± 0.162	^*^07 ± 0.044
		Size in *µ*m	90 ± 0.897	80 ± 1.333	^*^130 ± 1.112	^*^95 ± 1.211

*Catharanthus roseus *L.	Epidermal cells	Frequency	144 ± 1.125	117 ± 1.254	^*^128 ± 1.105	^*^100 ± 1.002
		Size in *µ*m	*L*—20 ± 0.789	*L*—14 ± 0.324	^*^ *L*—25 ± 0.771	*L*—18 ± 0.110
			*B*—14 ± 0.421	*B*—09 ± 0.211	^*^ *B*—17 ± 0.114	*B*—11 ± 0.098
	Stomata	Frequency	34 ± 0.457	12 ± 0.152	^*^29 ± 0.356	^*^08 ± 0.321
		Size in *µ*m	*L*—20 ± 0.789	*L*—18 ± 0.324	*L*—21 ± 0.771	*L*—20 ± 0.110
			*B*—12 ± 0.421	*B*—09 ± 0.211	*B*—13 ± 0.114	*B*—10 ± 0.098
		Stomatal index	20 ± 0.459	09 ± 0.103	^*^15 ± 0.451	^*^05 ± 0.357

*L*: length, *B*: breadth; data represent S.E. (*n* = 3). ^*^indicates values significant from healthy site (*P* ≤ 0.05).
